# Systemic sclerosis associated with primary sclerosing cholangitis and hyperthyroidism: a case report

**DOI:** 10.1097/MS9.0000000000000727

**Published:** 2023-05-03

**Authors:** Ali Ghassa, Hatem Kilani, Safa’a Al-Sayed, Tamim Alsuliman

**Affiliations:** a Faculty of Medicine, Damascus University; b Departments of Rheumatology, Alassad University Hospital, Damascus, Syria; c Service d’hématologie, hôpital Saint-Antoine, AP-HP Sorbonne Université, Paris, France

**Keywords:** hyperthyroidism, primary sclerosing cholangitis, Systemic sclerosis

## Abstract

The association between systemic sclerosis and other immune-mediated diseases such as primary sclerosing cholangitis and hyperthyroidism should be suspected in case the patient presents with similar symptoms. A 43-year-old woman presented to the hospital with progressive jaundice, dark urine, dyspnoea, fatigue, generalized arthralgia, weight loss, and amenorrhoea. In addition, she was diagnosed with systemic sclerosis seven years ago. The patient was diagnosed recently with primary sclerosing cholangitis and hyperthyroidism through laboratory tests and investigations such as magnetic resonance cholangiopancreatography, ultrasound, and computed tomography scan. After appropriate treatment and follow-up, the patient recovered well. Immune-mediated diseases can occur simultaneously or consequently due to the common immunological dysfunction that causes these conditions.

## Introduction

Highlights

- Immune-mediated diseases can occur simultaneously or consequently.
- The most common manifestation of systemic sclerosis is the dermatological one.
- There is no specific cure for systemic sclerosis, as it depends on curative methods for life-threatening conditions.
- Systemic sclerosis usually accompany primary biliary cirrhosis. However, primary sclerosing cholangitis is extremely rare to accompany it.
- The most common autoimmune disease to accompany systemic sclerosis is Hashimoto’s disease and Grave’s disease is less likely to occur with systemic sclerosis.

Systemic sclerosis (SSc) is a chronic inflammatory disease that affects multiple organs^1^. It is considered a relatively rare disease. Genetic, environmental and chemical exposure causes have a significant role in the aetiology. Diagnosis of SSc is made based on the combination of clinical manifestations including skin and other organs such as the lung, gastrointestinal (GI) tract, and kidney, and laboratory tests, including systemic sclerosis-related antibodies^2^.

Primary sclerosing cholangitis (PSC) is a chronic inflammatory disease that affects the liver and bile ducts leading to fibrosis and cirrhosis. The exact cause of PSC is still unknown, but autoimmune, environmental, and ischaemic factors might explain the aetiology^3^. Hyperthyroidism is a condition that happens when there are increased levels of free serum thyroxine (T4) or/and triiodothyronine (T3), leading to a hypermetabolic state in the body^4^. We present a rare case of systemic sclerosis followed by primary sclerosing cholangitis and hyperthyroidism. To our knowledge, it is the first case that describes this association.

## Case presentation

A 43-year-old woman presented to the hospital with progressive jaundice, dark urine, dyspnoea, fatigue, generalized arthralgia, and weight loss. The symptoms were progressing over the past year. She was also suffering from amenorrhoea for the last 6 months.

She was diagnosed 7 years ago with systemic sclerosis based on Raynaud’s phenomenon, GI symptoms (heartburn), and laboratory tests.

The patient does not use tobacco or alcohol and she is treated for hypertension. She mentioned having several toes of both feet amputated because of digital ulcers and ischaemia due to Raynaud’s.

Family history is insignificant for any relevant disease except for early menopause in two of the patient’s relatives.

Physical examination showed scleral icterus, pallor, telangiectasia and tight skin on both face and hands, with pitting sores on the fingers. She has second-grade pitting oedema, in addition to Soft crackles that were noticed in lung auscultation.

The vital signs were measured: (blood pressure: 160/100 mmHg, pulse rate: 110, respiratory rate: 20/min, and saturation O_2_: 96%).

Laboratory findings (shown in Table 1) showed elevated bilirubin on behalf of conjugated bilirubin and elevated alkaline phosphatase. These both are most likely to rise in biliary ducts diseases. Negative anti-smooth muscle antibody ruled out autoimmune hepatitis. Although antimitochondrial antibody is positive, it does not diagnose primary biliary cirrhosis since it is weakly elevated and can occur in several autoimmune diseases. Low thyroid stimulating hormone, high free thyroxine, and normal T3 are consistent with hyperthyroidism. Since follicle-stimulating hormone and prolactin is in the normal range, amenorrhoea is mostly a result of hyperthyroidism. We can exclude menopause due to normal follicle-stimulating hormone. Other causes of secondary amenorrhoea are less likely, noting that the patient is not married or sexually active.

**Table 1:** Patient's laboratory tests.

laboratory test	Laboratory result
Hb	11.6 mg/dl
Hematocrit	35%
RBC	3.7 × 10^6^
Mean corpuscular volume	93 fl
Mean Corpuscular Hemoglobin	30 pg
WBC	27 000 cells/mcl
PLT	213 000 mcl
Erythrocyte sedimentation rate	84 mm/h
Urea	42 mg/dl
Creatinine	0.6 mg/dl
Glucose	72 mg/dl
Cholesterol	468 mg/dl
Triglycerides	201 mg/dl
Total protein	6.5 g/dl
Albumin	3.3 g/dl
Total bilirubin	10.4 mg/dl
Conjugated bilirubin	8.2 mg/dl
Alkaline phosphatase	1146 U/l
Alanine transaminase	78 U/l
Aspartate aminotransferase	47 U/l
FSH	5 IU/l
TSH	0.06 mIU/l
FT4	2.38 ng/dl
T3	72 ng/dl
ANA	1:100
ASMA	Negative
AMA	1:10

ALT, Alanine transaminase; ANA, antinuclear antibody; AMA, antimitochondrial antibody, ASMA, anti-smooth muscle antibody; AST, Aspartate aminotransferase; ESR, Erythrocyte sedimentation rate; FSH, follicle-stimulating hormone; FT4, free thyroxine; Hb, haemoglobin; HCT, Hematocrit; MCV, Mean corpuscular volume; MCH, Mean Corpuscular Hemoglobin; PLT, platelet; RBC, red blood cell; T3, triiodothyronine; TSH, thyroid stimulating hormone; WBC, white blood cell.

**Figure 1:**
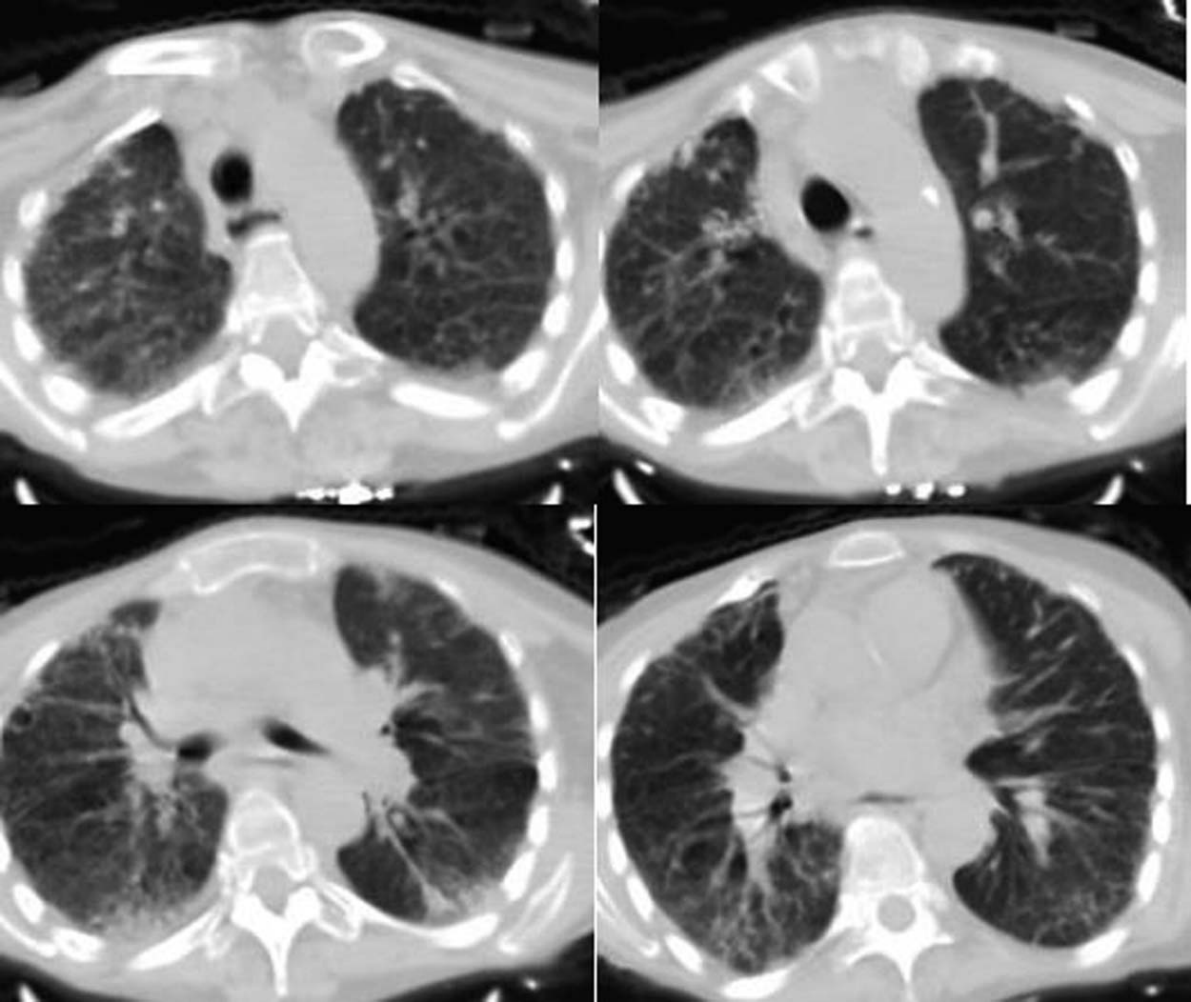
A chest CT scan, showing the interstitial pulmonary fibrosis. CT, computed tomography.

**Figure 2:**
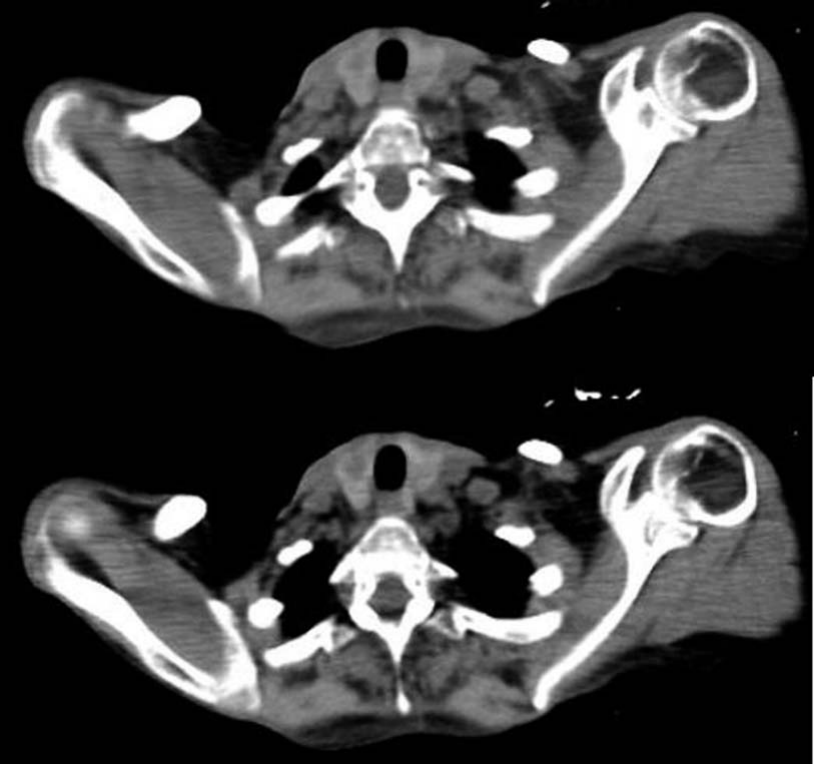
A CT scan, showing the multinodular appearance of the thyroid gland. CT, computed tomography.

Chest computed tomography and pulmonary function test confirmed interstitial fibrosis due to systemic sclerosis. (Fig. 1) Magnetic resonance cholangiopancreatography (MRCP) revealed multifocal stenosis in extrahepatic and intrahepatic bile ducts. The patient refused a liver biopsy to confirm the diagnosis accurately and a colonoscopy to detect ulcerative colitis since it is highly accompanied with PSC. The Ultrasound and the computed tomography scan of the thyroid gland demonstrated a multinodular appearance. (Fig. 2) MRCP and lab tests enabled us to diagnose PSC and exclude other conditions.

The patient is being treated with mycophenolate mofetil (1 g/day) for pulmonary manifestations, Hydroxychloroquine (400 mg/day) for arthralgias, Esomeprazole (20 mg/day) for gastroesophageal reflux and Pentoxifylline (800 mg/day) to improve blood flow in blood vessels. Ursodeoxycholic acid (750 mg/day) and Prednisolone (20 mg/day) were prescribed for PSC, in addition to Methimazole (10 mg/day) for hyperthyroidism. Endoscopic retrograde cholangiopancreatography and stent therapy was not performed for the patient.

The patient stayed at the hospital for four days after the diagnosis was made. The patient’s symptoms improved, and jaundice was less existed, then she was discharged upon her request. After six months, she told us that she is recovering well, with no jaundice remaining, but still not gaining weight or menstruating. Her doctor, who followed her in her city, told us that her lab tests are within the normal range.

## Discussion

SSc is a complex chronic autoimmune-mediated disease characterized by the fibrosis of the skin and multiple other organs^1^. The most common manifestation of SSc is the dermatological one presenting as skin sclerosis, Raynaud’s phenomenon with digital vasculopathy, pigmentation, calcinosis, and leg ulcers^5^. Raynaud’s phenomenon is usually the first manifestation of SSc, which results from abnormal digital perfusion. The common symptom is digital colour changes. The episode of Raynaud starts with white or pallor, followed by blue and finally red fingers. Progressive vasculopathy can lead to ischaemia and gangrene, and ends up in digital loss^6^.

The pulmonary demonstration is usually the mortality cause. It presents in the form of interstitial lung disease or pulmonary hypertension^7^. The association between SSc and the digestive system is very common. It can occur in any part from the mouth to the anus, but the oesophagus is the most common affected organ. Dysphagia, heartburn, dyspepsia, nausea, vomiting, abdominal bloating, and faecal incontinence are common symptoms in patients with GI tract involvement^8^.

According to the American College of Rheumatology/ European League against Rheumatism, there is a criteria containing skin manifestations and systemic sclerosis-related antibodies containing anti-centromere antibodies, anti-topoisomerase I (anti-Scl70) antibodies and anti RNA polymerase III antibodies, which the patient should meet to be diagnosed with SSc^2^.

There is no specific cure for SSc, as it depends on curative methods for life-threatening conditions. Treatment depends on the subtype of SSc. For early progressive cutaneous disease, methotrexate is the best choice, but for other dermatological conditions, for example, Raynaud’s phenomenon, lifestyle modification (smoking cessation and avoiding cold), and nitrates or calcium channel blockers or phosphodiesterase type 5 are the best treatments. (hughes 2020) Respiratory symptoms are treated with mycophenolate mofetil or rituximab. Angiotensin-converting enzyme inhibitors are used for sclerodermal renal crisis^2,9^.

PSC is a chronic inflammatory disease that affects the liver and biliary ducts, causing fibrosis and multifocal stenosis to them. PSC patients can be asymptomatic or have some symptoms such as fatigue, pruritis, and jaundice. It is diagnosed through MRCP, which shows a mixture of multifocal strictures and saccular dilatations of the bile ducts, known as (“beaded” appearance). Increased alkaline phosphatase 3–5 times normal can be seen in laboratory tests^3^. PSC treatment consists of symptoms treatments, cholestyramine for pruritis, antibiotics in case of cholangitis development, and vitamin D for bone disease (e.g. osteoporosis). Balloon dilatation or plastic stent insertion in bile ducts can also be done. Surgery decision (extrahepatic bile duct resection) is made when endoscopic treatment and liver transplant are not available^10^.

SSc is usually accompanied with primary biliary cirrhosis. However, its association with PSC is extremely rare^11^. The exact reason for the association between SSc and PSC is unknown. Variable theories can explain this causality. One of these is the similarity of major histocompatibility complex MHC types among SSc and PSC patients. SSc is usually accompanied with HLA-B8 and DR3, while PSC is associated with HLA DR3^12^. Another theory suggests that the relationship between these two disorders is caused by the elevated interleukin-8 in patients’ serum^13^.

Hyperthyroidism is a disease characterized by the overproduction of thyroidal hormones from the thyroid gland^4^. The most common cause of hyperthyroidism is grave’s disease^14^. Hyperthyroidism often presents with fatigue, sweating, heat intolerance, weight loss, and amenorrhoea in female patients. Suppressed thyroid stimulating hormone with high levels of free thyroxine or free triiodothyronine can confirm the diagnosis^14^. Hyperthyroidism treatment options contain antithyroid drugs such as propylthiouracil or methimazole, radioactive iodine therapy, and surgery such as total or subtotal thyroidectomy. In addition to beta-blockers for symptomatic thyrotoxicosis^14^.

Ferrari *et al*.^15^ mentioned in their paper multiple cases of grave’s disease associated with systemic sclerosis. Autoimmune thyroid diseases can occur with SSc, and the most common one is Hashimoto’s, while grave’s is less likely to accompany SSc. Unfortunately, few studies clarified the relationship between grave’s and SSc. In the Caucasian community, HLA-B8 and HLA-DR3 can be found in both diseases. In addition, various genes including HLA-DRB1, DQBI, and CTLA-4 are also involved in both conditions^16^.

There is a hypothesis indicating that the influence of environmental and genetic factors can determine autoimmune phenomena in multiple organs, characterized by the predominance of Th1 immune pattern at the initial or active phase of these disorders^15^.

There are some limitations of this case, for example, neither a liver biopsy was done to confirm PSC, nor radioactive iodine uptake for hyperthyroidism.

## Conclusion

In conclusion, immune-mediated diseases can occur simultaneously or consequently based on the common immunological dysfunction that causes these diseases. Thorough clinical examination is the key tool to detect such association, as indeed a train can hide another.

## Ethical approval

Not applicable

## Patient consent

Written informed consent was obtained from the patient to publish this report in accordance with the journal’s patient consent policy.

## Source of funding

None.

## Author contribution

A.G. gathered the data, researched the literature, and wrote the first draft. H.K. and S.A. treated and followed the patient and reviewed the article for scientific adequacy. T.A. revised the manuscript. All authors reviewed and approved the final manuscript before submission.

## Conflicts of interest disclosure

The authors declare that there is no conflict of interest to be reported.

## Research registration unique identifying number (UIN)

Not applicable.

## Guarantor

Ali Ghassa.

## Provenance and peer review

Not commissioned, externally peer-reviewed.

## Data availability statement

All data generated during this study can be accessed through direct communication with the corresponding author and the agreement of all research team members.
